# Chronic intermittent hypoxia enhances glycinergic inhibition in nucleus tractus solitarius

**DOI:** 10.1152/jn.00241.2022

**Published:** 2022-11-02

**Authors:** Shuping Jia, Nataliya Rybalchenko, Kishor Kunwar, George E. Farmer, Joel T. Little, Glenn M. Toney, J. Thomas Cunningham

**Affiliations:** ^1^Department of Physiology and Anatomy, University of Texas Health Science Center, Fort Worth, Texas; ^2^Microscopy Core, Division of Research and Innovation, University of Texas Health Science Center, Fort Worth, Texas; ^3^Department of Cellular & Integrative Physiology, University of Texas Health San Antonio, San Antonio, Texas

**Keywords:** astrocytes, chemoreceptor, GABA, protein trafficking

## Abstract

Chronic intermittent hypoxia (CIH), an animal model of sleep apnea, has been shown to alter the activity of second-order chemoreceptor neurons in the caudal nucleus of the solitary tract (cNTS). Although numerous studies have focused on excitatory plasticity, few studies have explored CIH-induced plasticity impacting inhibitory inputs to NTS neurons, and the roles of GABAergic and glycinergic inputs on heightened cNTS excitability following CIH are unknown. In addition, changes in astrocyte function may play a role in cNTS plasticity responses to CIH. This study tested the effects of a 7-day CIH protocol on miniature inhibitory postsynaptic currents (mIPSCs) in cNTS neurons receiving chemoreceptor afferents. Normoxia-treated rats primarily displayed GABA mIPSCs, whereas CIH-treated rats exhibited a shift toward combined GABA/glycine-mediated mIPSCs. CIH increased glycinergic mIPSC amplitude and area. This shift was not observed in dorsal motor nucleus of the vagus neurons or cNTS cells from females. Immunohistochemistry showed that strengthened glycinergic mIPSCs were associated with increased glycine receptor protein and were dependent on receptor trafficking in CIH-treated rats. In addition, CIH altered astrocyte morphology in the cNTS, and inactivation of astrocytes following CIH reduced glycine receptor-mediated mIPSC frequency and overall mIPSC amplitude. In cNTS, CIH produced changes in glycine signaling that appear to reflect increased trafficking of glycine receptors to the cell membrane. Increased glycine signaling in cNTS associated with CIH also appears to be dependent on astrocytes. Additional studies will be needed to determine how CIH influences glycine receptor expression and astrocyte function in cNTS.

**NEW & NOTEWORTHY** Chronic intermittent hypoxia (CIH) has been used to mimic the hypoxemia associated with sleep apnea and determine how these hypoxemias influence neural function. The nucleus of the solitary tract is the main site for chemoreceptor input to the CNS, but how CIH influences NTS inhibition has not been determined. These studies show that CIH increases glycine-mediated miniature IPSCs through mechanisms that depend on protein trafficking and astrocyte activation.

## Graphical abstract

**Figure:**
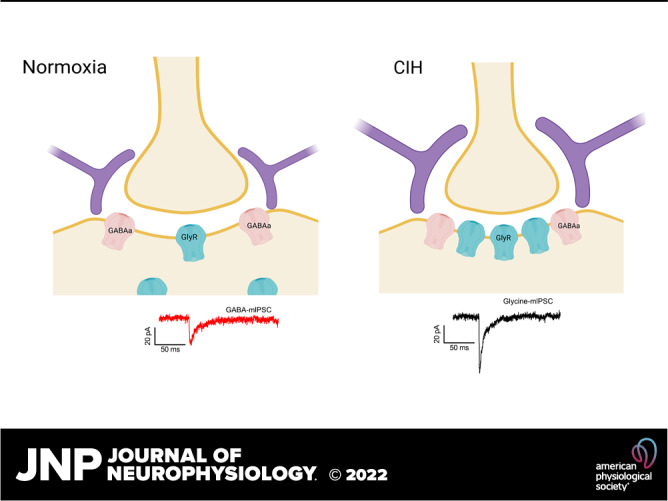

## INTRODUCTION

The nucleus tractus solitarius (NTS) is the primary integration center for afferent information arising from the arterial baroreceptors and chemoreceptors (1). Chronic intermittent hypoxia (CIH), an experimental animal model for studying impacts of hypoxemias related to sleep apnea (2), produces autonomic and cardiorespiratory alterations that lead to systemic hypertension (3, 4). Sleep apnea and the associated hypertension, are observed mostly in males although incidence in females increases following menopause (5, 6). Previous studies have shown that exposing adult male rats to 7 days of CIH is sufficient to produce sustained hypertension that is maintained throughout the entire diurnal cycle even when the rats are breathing room air (7). The increase in blood pressure associated with this model of CIH is not observed in gonadally intact female rats (8). Furthermore, the sustained hypertension associated with this model in males is dependent on changes in gene expression and on noradrenergic neurons in the NTS (9, 10).

Accumulated evidence indicates that CIH enhances afferent activity from arterial chemoreceptors and results in long-term synaptic plasticity within the NTS (11–15). And although numerous studies report that CIH influences excitatory neurotransmission in the NTS (16–18), limited information is available concerning the impact of CIH on inhibitory neurotransmission, a key determinant of chemosensitive NTS neuronal activity (19–21).

In the adult NTS, synaptic inhibition is mediated both by γ-aminobutyric acid (GABA) and glycine, with a predominance of GABA inhibition (15). In some systems, GABA and glycine are purportedly coreleased from the same synaptic vesicles (22, 23) and may be coexpressed in axon terminals throughout the NTS (22, 24, 25). However, effects of CIH on glycinergic inhibitory transmission involving rapid hyperpolarization due to Cl^−^ influx through ionotropic glycine receptors (GlyRs) (23) have not been established. Membrane expression of GlyRs and their trafficking are modifiable, which allows glycine to contribute to changes in neuronal function (26). To date, the possible role of glycine and GyRs in CIH-induced changes in NTS function has not been determined.

Recently, an increasing number of studies have implicated astrocytes in synaptic plasticity in NTS and control of cardiovascular function (27–31). Glial fibrillary acidic protein (GFAP), a commonly measured endogenous astrocyte-specific protein, is a validated marker of reactive astrocytes (32). GFAP expression dynamically upregulates in response to many local influences in the brain microenvironment (e.g., infection, injury, stress), resulting in structural changes. For example, GFAP is increased in NTS astrocytes following the initial phase (24 h) of hypoxia, or hypertension induced by hypoxia (30, 31). Rats with NTS astrocyte inactivation show large variations in arterial pressure, consistent with compromised cardiovascular reflex control (27). Recent studies demonstrate that NTS astrocytes contribute to tripartite synapses critical to control glutamatergic and GABAergic signaling (28, 29). Based on these reports, we tested the contribution of astrocytes to CIH-induced changes in glycinergic function.

## MATERIALS AND METHODS

### Animals

Adult male and female Sprague-Dawley rats (250–400 g) from Charles River Laboratories (Wilmington, MA) were individually housed in a temperature‐controlled environment with lights on from 7 AM to 7 PM cycle. All experiments were performed in accordance with National Institutes of Health guidelines and with the approval of the Institutional Animal Care and Use Committee of the University of North Texas Health Science Center (Fort Worth, TX).

### Carotid Body Anterograde Tracer Labeling Surgery

All rats were anesthetized with ketamine (75 mg/kg ip; Fort Dodge Laboratory) and Dexdomitor (0.5 mg/kg, ip Zoetis) to label carotid sinus nerve afferents as previously described (33). The anterograde fluorescent tracer DiA [4-Di-16-ASP (4-(4-(Dihexadecylamino) styryl)-N-Methylpyridinium Iodide; D3883, ThermoFisher, Waltham, MA] was applied unilaterally to the carotid body region and sealed in place with Kwik-Sil Silicone Adhesive (World Precision Instruments, Sarasota, FL). Atipamezole (Antisedan, 1 mg/kg ip; Pfizer Animal Health, New York, NY) was given to terminate anesthesia after surgery and the anti-inflammatory Rimadyl was administered after surgery. One week after the surgery, rats were exposed to either CIH or room air for 7 days.

### Chronic Intermittent Hypoxia Protocol

Rats that previously underwent surgery to label carotid sinus nerve terminals in NTS were housed in hypoxia chambers with ad libitum food and water access as previously described (34, 35). Chamber O_2_ concentration was controlled by a computerized system (Oxycycler, Biospherix, Parish, NY). The protocol used alternating 3-min cycles of 21% and 10% O_2_ for 10 cycles per hour for 8 light phase (8:00 AM to 4:00 PM) hours for 7 days. For the remaining 16 h each day, animals breathed normoxic room air. Control rats continuously breathed room air.

### Electrophysiology Preparation

Following normoxia or CIH exposure, rats were anesthetized with isoflurane (2–3%), perfused transcardially with cold-modified artificial cerebrospinal fluid (modified aCSF in mM: 103 sucrose, 63 NaCl, 26 NaHCO_3_, 1.25 NaH_2_PO_4_, 3 KCl, 0.5 CaCl_2_, 2.5 MgCl_2_, 2 MgSO_4_, 10 d-glucose) equilibrated with Carbogen (95% O_2_, 5% CO_2_), and their hindbrains were isolated. Coronal slices containing caudal NTS (250 µM thick) were cut in a MicroSlicer Zero 1 (Ted Pella Inc.) using a sapphire knife (Ted Pella Inc.). For protective recovery, slices were transferred to an incubation chamber containing prewarmed (34°C) aCSF composed of (in mM) 126 NaCl, 3 KCl, 1.25 NaH_2_PO_4_, 26 NaHCO_3_, 2 CaCl_2_, 2 MgSO_4_, and 10 d-glucose (300 mosmol/L) for ∼30 min. Slices remained in the recovery chamber at room temperature for another 1 h before proceeding with patch-clamp recordings. Incubation solutions were saturated with carbogen at least 10 min before use to ensure stable pH buffering (pH 7.3) and adequate oxygenation.

**Figure 1.**
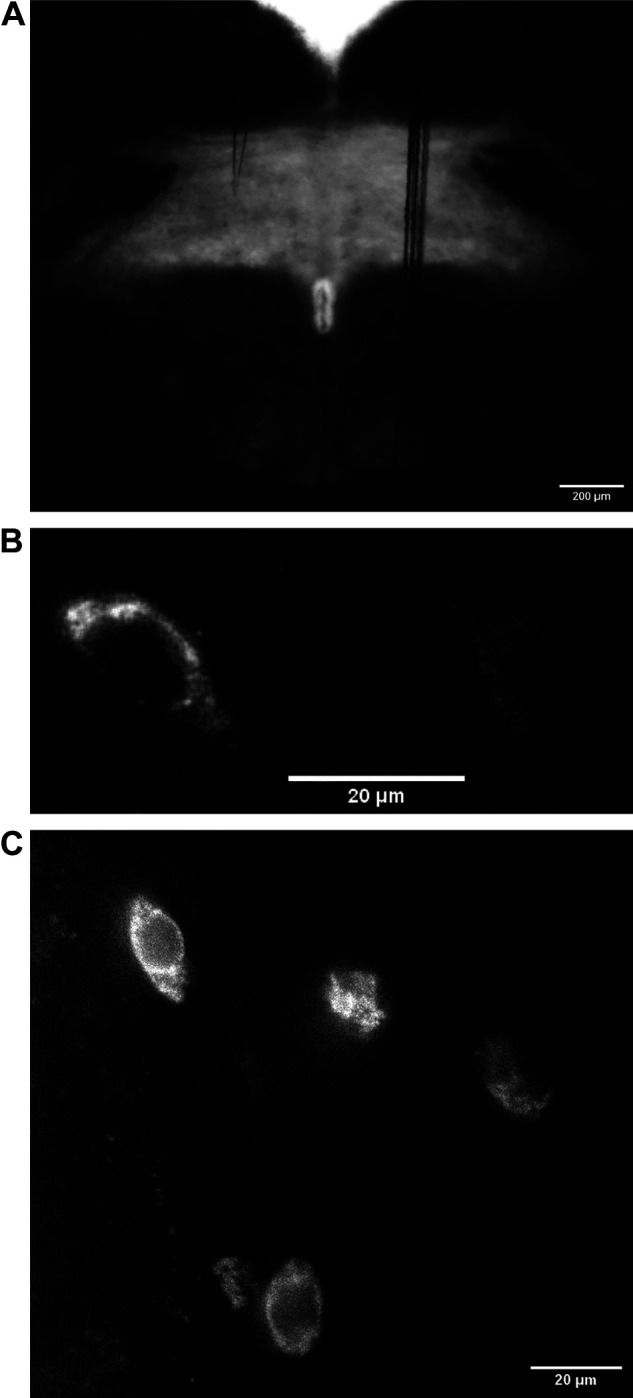
Digital images showing *A*: a coronal brain slice containing the caudal NTS where most of the recordings were obtained. *B*: representative image showing DiA fluorescent puncta from the carotid body associated with caudal NTS neuron. *C*: lower power magnification of several caudal NTS neurons in the same field showing DiA-labeled puncta. DiA, 4-Di-16-ASP (4-(4-(Dihexadecylamino) styryl)-*N*-methylpyridinium iodide; NTS, nucleus tractus solitarius.

#### Whole cell patch-clamp recording.

Each hindbrain slice was transferred from the recovery chamber to a recording chamber and perfusion of oxygenated (5% CO_2_-95% O_2)_ aCSF at room temperature. Receptor antagonists were bath applied at a flow rate of 2–2.5 mL/min. Whole cell voltage-clamp recordings were performed using a MultiClamp 700B amplifier (Molecular Devices, CA). Recordings were obtained from putative second-order NTS neurons identified by DiA labeling (Fig. 1). In other experiments, recordings were obtained from neurons in the dorsal motor nucleus of the vagus (DMNX). A horizontal micropipette puller (P-97 Foaming Brown, Sutter Instruments, Novato, CA) was used to fabricate recording electrodes from borosilicate glass capillaries (2–5 MΩ), which were filled with a solution containing (in mM): 70 K-gluconate, 70 KCl, 2 NaCl, 10 HEPES, 4 EGTA, 4 Mg-ATP, and 0.3 Na2-GTP.

#### Miniature postsynaptic current recordings.

Miniature inhibitory postsynaptic currents (mIPSCs) were recorded in voltage-clamp mode (Vhold: −60 mV) from labeled NTS neurons in slices prepared from normoxic or CIH rats. Currents were low-pass-filtered at 2 kHz and sampled at 10 kHz. Each recording was performed in the presence of 6-cyano-7-nitroquinoxaline-2,3-dione (CNQX, 10 µM, Tocris, Minneapolis, MN) to block AMPA receptors and tetrodotoxin (TTX, 1 µM, Tocris) to block Na^+^ channel-mediated action potentials, thereby eliminating excitatory glutamatergic inputs. mIPSCs were collected over a 4 min baseline period and continually recorded for another 5 min after addition of bath SR95531 (25 µM, Tocris) to abolish GABA_A_ receptor (GABA_A_R)-mediated currents and isolate GlyR-mediated mIPSCs. In some experiments, strychnine (3 µM Tocris) was used to block glycine currents. Our recording conditions (heightened intracellular chloride concentration of 72 mM, ECl^−^ = approximately −15 mV) made glycinergic and GABAergic currents appear inward at our holding potential of −60 mV.

#### Brefeldin-A application.

To test the role of protein trafficking in the CIH-induced modulation of GlyR currents, brain slices were incubated with 5 µM brefeldin-A (BFA) for 20 min before recording mIPSCs from putative second-order chemoreceptor neurons. After the 20 min incubation, slices were transferred to the recording chamber and perfused with an aCSF solution containing 10 µM CNQX and 1 µM TTX, but with BFA omitted. After a 4 min baseline recording, 25 µM SR95531 and 3 µM strychnine were added to the bath solution.

#### Fluorocitrate application.

In some experiments, mIPSCs were recorded in the presence of either fluorocitrate (FC, 100 µM), an inhibitor of astrocyte metabolism, or vehicle (Veh). Stock FC solution (100×) was prepared as follows:  100 mg of d,l-fluorocitric acid barium salt (Sigma Aldrich) was dissolved in 2.5 mL of 0.5 mM HCl. Next, 100 µL of Na_2_SO_4_ was added to precipitate the Ba^2+^ followed by 5 mL of 0.5 mM Na_2_HPO_4_. The suspension was centrifuged at 1,000 *g* for 5 min. Supernatant was diluted with aCSF (without CaCl_2_) to 10 mM final concentration (total volume was 12.1 mL), and pH was adjusted to 7.4. This FC stock solution was aliquoted and stored in −20°C. Slices were preincubated in aCSF containing 100 µM FC for 2 h and perfused with the same solution during recording. Vehicle solution contained the same concentrations of HCl, Na_2_SO_4_, and Na_2_HPO_4_ that were diluted in aCSF and adjusted to pH 7.4.

### Western Blot Analysis

Fresh coronal slices containing caudal NTS (300-µm thick) were prepared from 300–500 g male rats (4 rats/group) and incubated with 100 µM FC or Veh for 2 h (the same procedure as for mIPSCs recording). Caudal NTS was dissected from surrounding tissue and each was separately homogenized in the ice-cold Radio-Immunoprecipitation Assay homogenization buffer, sonicated, and centrifuged at 30,000 *g* for 20 min. Supernatant was collected and protein concentration was measured by bicinchoninic acid assay (Pierce, Cat. No. 23225). Protein (25 µg) from each sample was mixed with 6 µL of SDS-loading buffer dye and boiled for 5 min. Protein samples were subjected to SDS-polyacrylamide gel electrophoresis (SDS-PAGE) and transferred to PVDF membranes. After blocking with 5% nonfat milk at room temperature, membranes were incubated with rabbit polyclonal anti-GFAP primary antibody (1:5,000, Abcam, ab7260, Waltham, MA) overnight at 4°C, followed by incubation with horseradish peroxidase-conjugated goat anti-rabbit IgG secondary antibody (1:10,000, Invitrogen, No. 31460) for 1 h at room temperature. Blots were visualized by enhanced chemiluminescence. Norm-Veh and CIH-Veh were set to 100%. Changes of GFAP/β-actin GeneTex, (GTX629630, Irvine, CA) ratio as a function of FC (100 µM) incubation were compared with their vehicle control and shown as a percentage of control intensity.

### Immunofluorescence and Confocal Microscopy

Immunofluorescence to visualize glycine receptor expression in NTS neurons was performed in three normoxic rats and three CIH rats anesthetized with isoflurane (2–3%) and perfused transcardially with PBS followed by 4% paraformaldehyde (PFA). Hindbrains were postfixed in 4% PFA for 24 h. After fixation, brains were dehydrated in 30% sucrose at 4°C. Brainstems were serially cut in coronal sections (20-μm thick) on a Leica CM 1950 cryostat and stored in cryoprotectant at −20°C.

After incubation for 2 h in blocking buffer containing 10% normal horse serum (NHS), sections were incubated with a primary antibody raised against a recombinant protein that corresponds to amino acids 1–457 of the α1 subunit of the rat glycine receptor (mouse anti-GlyR antibody, 1:500, Synaptic Systems, 146-011, Goettingen, Germany) and with either an anti-NeuN antibody, (rabbit anti-NeuN antibody, 1:500, Abcam, ab 177487) or an anti-GFAP antibody (rabbit anti-GFAP antibody, 1:500, Abcam, ab68428) for 2 days at 4°C. Next, the sections were incubated with appropriate secondary antibodies conjugated with either Alexa Fluor 488 (donkey anti-mouse, 1:500, Jackson ImmunoResearch Laboratories 715-545-151) or Alexa Fluor 594 (donkey anti-rabbit, 1:500, Jackson ImmunoResearch Laboratories 711-585-152) for 4 h at room temperature. Stained sections were mounted on gelatin-coated slides and coverslipped using ProLong Gold mounting media (Thermo Fisher). Control sections were processed using the same protocol with the omission of the primary antibodies. No staining was observed in any of these sections.

Confocal images were acquired on an LSM 880 Airyscan Confocal Microscope and samples were illuminated sequentially with 488-nm Argon and 561-nm HeNe lasers at 1,000 and 60 µW, respectively. Images were acquired using appropriate filter sets (BP 420–480 + BP 495–550) and (BP 570–620 + LP 645) and were processed with three-dimensional (3-D) Airyscan processing function of Zen black software. FIJI/Image J and 3-D ImageJ suite were used for further processing and quantification (36, 37). 3-D segmentation outlines of the cells based on the NeuN fluorescence signal were performed semiautomatically using the 3-D ImageJ suite. 3-D segmentations of GlyR-immunoreactive puncta were performed using a custom-macro in FIJI. Briefly, background subtraction was performed with a rolling ball radius = 15, min and max were set to 80 and 2,000, respectively, on the 16-bit image and converted to 8-bit. 3-D simple segmentation used low threshold = 15, min size = 10, max size = −1. Segmented 16-bit images were converted to 8-bit binary and used for analysis. Binary images of cell segmentation and GlyR-positive puncta segmentations inside the cell outline were added to RoiManager3D v3.96 from which total voxel volumes of each were calculated. Only fully captured cells were used for analysis. Partially captured cells on the image frame were removed manually. GlyR staining on NTS neurons was quantified as the relative volume of GlyR puncta associated with the cell normalized by the whole cell volume (percentage of GlyR puncta per cell = volume of the GlyR puncta inside the cell/volume of the cell segmentation*100).

### Data Analysis

mIPSCs were analyzed off-line using Synaptosoft Mini Analysis Program (v. 6.0.3; Synaptosoft, Decatur, GA). All mIPSCs traces and cumulative probabilities were plotted by Igor Pro 4.0. Differences were analyzed for statistical significance by one-way ANOVA followed by Tukey’s honest significance test (GraphRobot Lei A. Wang, 2019, https://www.graphrobot.com/; GraphPad Prism version 9.1.1 for Windows, GraphPad Software, San Diego, CA, www.graphpad.com). Contingency table tests were performed using Prism. A level of *P* < 0.05 was required for significance. Unless otherwise stated, all data are expressed as the means ± SE. Student’s unpaired *t* test was used to compare statistical significance between unexposed and CIH-exposed groups.

## RESULTS

### CIH Increases NTS Glycinergic mIPSCs

Our experiments focused on the areas of commissural, medial, and dorsomedial subnuclei of the caudal NTS adjacent to the tractus, which are primary targets for afferent input originating from carotid body chemoreceptors (38, 39). Baseline mIPSCs were recorded from DiA-labeled second-order NTS neurons prepared from normoxic (*n* = 12) and CIH (*n* = 8) rats by using the whole cell voltage-clamp technique with Vm held at −60 mV for a 4 min post break-in period and a bath solution containing 1 µM TTX and 10 µM CNQX. There was a significant difference in holding current for neurons from normoxic controls compared with CIH-exposed rats [Norm −16.24 ± 2.9 pA; CIH −5.3 ± 2 pA; *t*(79) = 3.1, *P* = 0.0024]. CIH did not affect cell capacitance [Norm 35.2 ± 1.8 pF; CIH 40.5 ± 2.6 pF; *t*(78) = 1.67, *P* = 0.0983].

**Figure 2.**
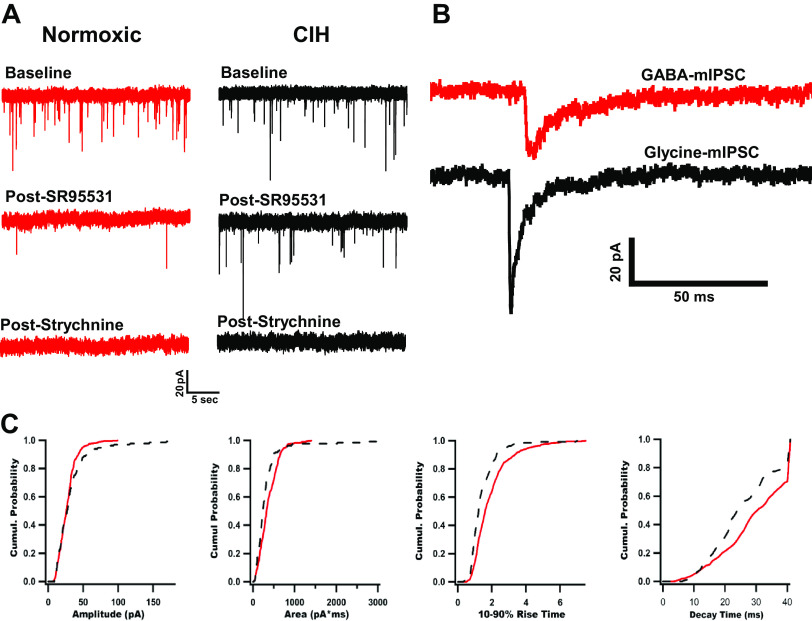
Spontaneous miniature inhibitory postsynaptic currents (mIPSCs) in second-order medial NTS neurons receiving arterial chemoreceptor inputs. *A*: representative dual GABA/Gly mIPSCs in NTS neurons from normoxic (red) and CIH (black) exposed rats (*left*). *B*: expanded time scale (*right*) illustrates amplitude and kinetic differences. *C*: cumulative probabilities of the baseline mIPSCs plotted from *A*. CIH, chronic intermittent hypoxia; NTS, nucleus tractus solitarius.

Some cells had inward mIPSCs that were a mixture of GABAergic and glycinergic currents, hereafter called dual GABA/Gly mIPSCs, while others showed only GABA mIPSCs. To estimate the relative proportion of glycine, GABA, and dual events, glycinergic or GABAergic mIPSCs were subsequently isolated by bath applying 25 µM SR95531 (gabazine) alone or SR95531 followed by 3 µM strychnine. Representative dual GABA/Gly mIPSC traces of cells from a normoxic and CIH rat are shown in Fig. 2, *A* and *B*. GABA-mediated mIPSCs were the major contributor to the total mIPSC activity in the normoxic cell. By contrast, the incidence of glycinergic events increased significantly in the cell from the CIH rat. The cumulative distribution of amplitudes, areas, 10–90% rise time, and decay time were obtained from each cell (Fig. 2*C*). The cumulative distribution of mIPSCs from CIH cells was shifted to larger amplitudes and areas, shorter rise times, and a trend for shorter decay times as compared with cells from normoxic rats. These differences were not observed in mIPSCs recorded from slices prepared from female rats exposed to 7 days of CIH as compared to recordings obtained from female normoxic control rats (amplitude Norm −26.37 ± 1.06 pA (*n* = 20 cells from 3 rats); CIH −35.59 ± 3.53 pA (*n* = 23 cells from 3 rats); *t*(41) = 1.898, *P* = 0.0647; Area Norm 313.3 ± 17.52 pA*ms (*n* = 18); CIH 371.2 ± 18.40 (*n* = 21); *t*(37) = 1.706, *P* = 0.0964; Rise time Norm 1.482 ± 0.07 ms (*n* = 20) CIH 1.493 ± 0.06 ms; t(41) = 0.09429, *P* = 0.9253; decay Norm 23.04 ± 1.1 ms (*n* = 20); CIH 20.83 ± 0.76 ms (*n* = 23); *t*(41) = 1.708, *P* = 0.0952).

**Figure 3.**
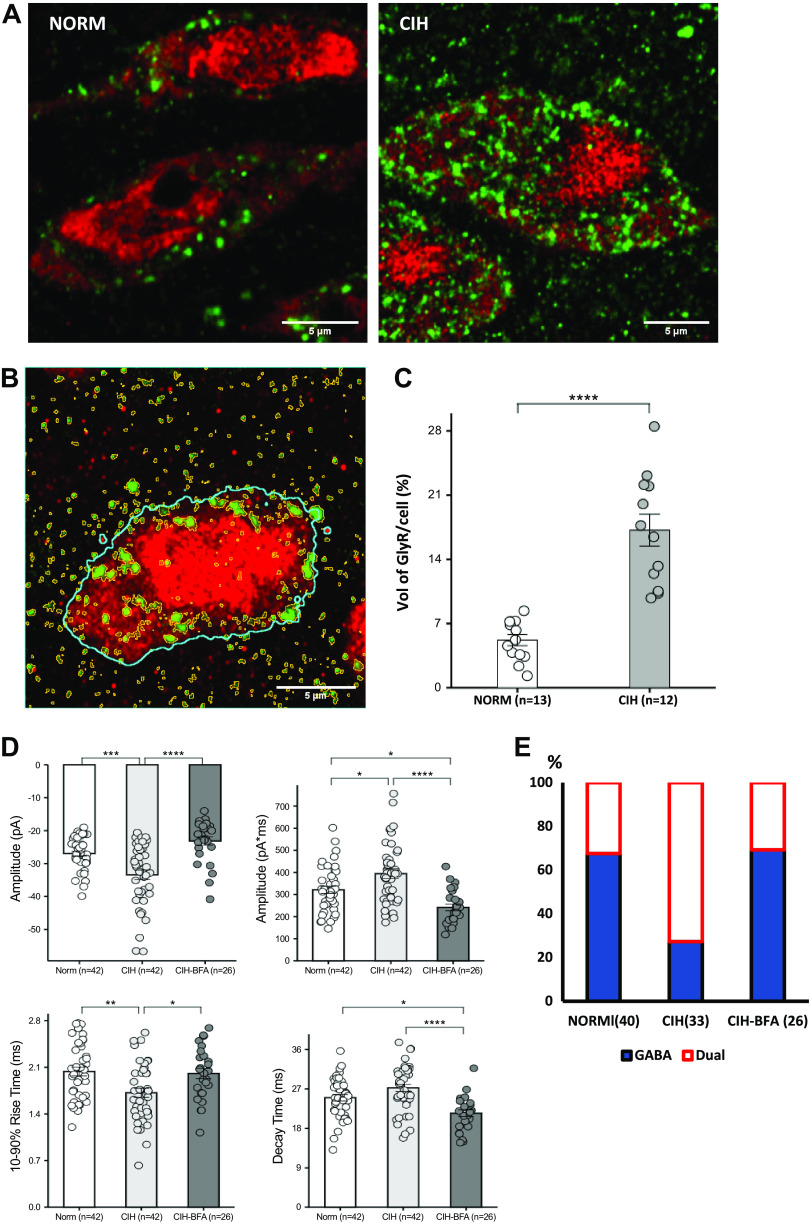
CIH-associated GlyR expression upregulation and GlyR-mediated mIPSC enhancement in NTS neurons was blocked by brefeldin A (BFA), an inhibitor of intracellular protein trafficking. *A*: representative confocal images showing increased GlyR (green) distribution on NTS neurons (red, anti-NeuN) from CIH compared with normoxic rats. *B*: illustration of voxel-size of GlyRs (all yellow ROIs) and whole cell volume in a representative NTS neuron (blue outline) from all Z-stack sections. *C*: percentage of the total GlyR volume (sum of the ROIs) vs. whole cell volume calculated and compared between normoxic and CIH groups showing that GlyR expression was significantly increased among individual neurons from CIH-exposed rats. *D*: increases of mIPSC amplitude and area, and their faster rise time after CIH were significantly attenuated by preincubation of slices with 5 µM BFA. BFA also increased decay kinetics compared with mIPSCs obtained from normoxic and CIH cells. *E*: the increase of dual GABA/Gly components associated with CIH (72.72%) was significantly reversed by pretreatment with BFA (30.7%). *P < 0.05. **P < 0.005. ***P < 0.001. ****P < 0.0001. CIH, chronic intermittent hypoxia; mIPSC, miniature inhibitory postsynaptic current; NTS, nucleus tractus solitarius. For *C*–*E*, each *n* represents the numbers of cells.

The effect of CIH on GlyR expression and membrane localization was determined from confocal images and 3-D reconstructions. Distribution of GlyRs on NTS neurons from rats exposed to normoxia or CIH (Fig. 3*A*) revealed that CIH markedly increased GlyRs [*t*(23) = 6.7, *P* < 0.0001] based on the percentage of voxel volume of GlyR-positive puncta relative to that of the cell (Fig. 3*B*). Results suggest that GlyR expression was markedly upregulated after 7 days of CIH compared with normoxic conditions (Fig. 3*C*).

### Brefeldin-A Blocks CIH Increase of NTS Glycinergic mIPSCs

mIPSCs from slices prepared from CIH-treated (*n* = 42) rats showed increased peak amplitude and area with faster 10–90% rise time (Fig. 3*D*) compared with those from normoxic rats (*n* = 42). Effects of CIH were not observed in slices incubated with brefeldin-A (BFA, *n* = 26). Average mIPSC amplitude in the CIH group was significantly increased compared with the normoxic group and this change was blocked by BFA [*F*(2,109) = 17.64, *P* < 0.0001; Norm vs. CIH, *P* < 0.0003; CIH vs. CIH + BFA, *P* < 0.0001]. BFA likewise prevented the increase of mIPSC area by CIH [*F*(2,109) = 14.59, *P* < 0.0001; Norm vs. CIH, *P* < 0.0105; CIH vs. CIH + BFA, *P* < 0.0001; Norm vs. CIH + BFA, *P* = 0.0012]. CIH exposure increased mIPSC onset kinetics, significantly decreasing rise time [*F*(2,109) = 7.112, *P* < 0.0012; Norm vs. CIH, *P* < 0.0019; CIH vs. CIH + BFA, *P* < 0.0160). Though unaffected by CIH, mIPSC decay time was significantly reduced by BFA [*F*(2,109) = 10.99, *P* < 0.0001; Norm vs. CIH + BFA, *P* < 0.0172; CIH vs. CIH + BFA, *P* < 0.0001]. Most neurons from CIH rats (24/33) had both SR95531- and strychnine-sensitive mIPSCs. This increase in the fraction of neurons with dual mIPSCs was statistically significant compared with normoxic controls [χ^2^ (1) = 11.7, *P* = 0.0006; Fig. 3*E*], and this shift was blocked by BFA. Results suggest that CIH is associated with an increase in GlyR expression in second-order chemoreceptor neurons in caudal NTS that result in mIPSCs with larger amplitude, greater charge transfer, and faster onset kinetics that reflect CIH-induced increase in protein expression/membrane trafficking.

### CIH Does Not Affect mIPSPs in DMNX

**Figure 4.**
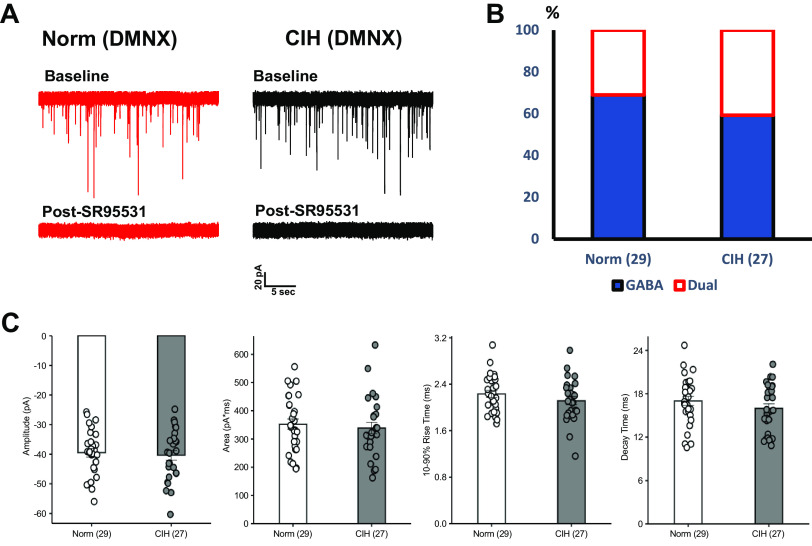
CIH induced changes in glycinergic mIPSCs are absent in neurons from the dorsal motor nucleus of the vagus (DMNX). *A*: representative mIPSCs recorded from DMNX neurons showing that SR95531 abolished almost all mIPSCs, although some cells had sparse strychnine-sensitive mIPSCs. *B*: no significant difference was seen in the proportion of dual GABA/Gly mIPSCs between each group. *C*: CIH did not significantly affect either mIPSC amplitude, area, 10–90% rise time or decay time of DMNV neurons. CIH, chronic intermittent hypoxia; mIPSC, miniature inhibitory postsynaptic current. Each *n* represents the numbers of cells.

Whole cell voltage-clamp was used to record mIPSC from the DMNX, adjacent to the NTS, in brain slices prepared from rats exposed to normoxia or CIH (Fig. 4*A*). In most cells, all mIPSCs were blocked by bath application of SR95531 and few cells had strychnine-sensitive mIPSCs (Fig. 4*B*). In contrast to neurons from the NTS, CIH did not increase the number of DMNX neurons with strychnine-sensitive mIPSCs (Fig. 4*B*) or significantly change any of the parameters of the mIPSCs (Fig. 4*C*). Together these results indicate that CIH did not affect mIPSCs in DMNX and the effects of CIH on glycinergic neurotransmission appear specific to the NTS.

**Figure 5.**
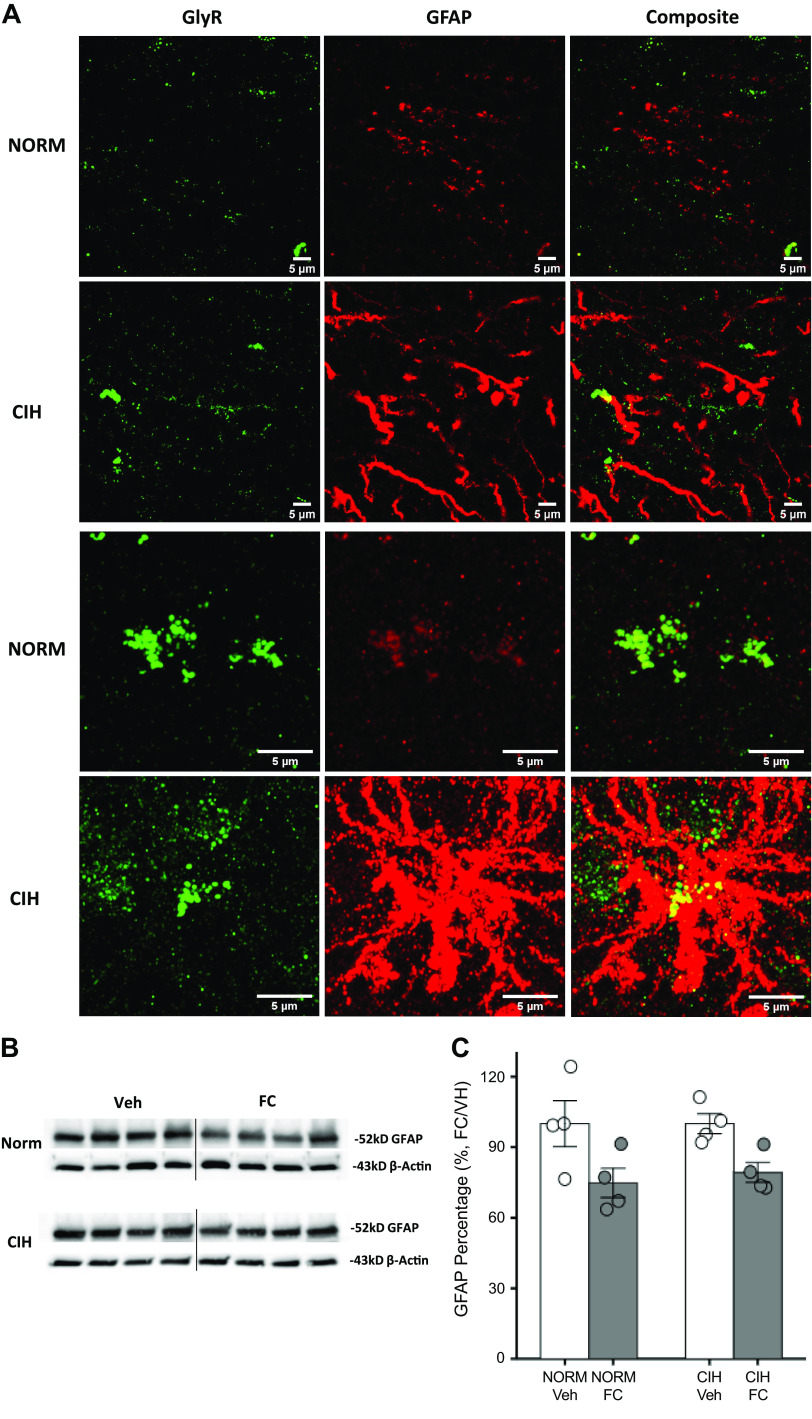
CIH increases glial fibrillary acidic protein (GFAP) in NTS astrocytes. *A*: confocal images showing GFAP (red, Alexa Fluor 594) and GlyR (green, Alexa Fluor 488) from normoxic and CIH NTS samples (*top* pair of images from single slices; *bottom* pair were the maximum intensity projection from corresponding Z-stacks). Elevated GFAP expression and astrocyte morphological changes were seen in CIH tissue samples. *B*: GFAP protein levels were compared by Western blot between 100 µM fluorocitrate- (FC) and vehicle (Veh)-treated caudal NTS tissue samples from normoxic (*top*) and CIH (*bottom*) rats (4 rats/group). *C*: summary data showing that FC decreased GFAP abundance in both treatment groups, i.e., not CIH specific [two-way ANOVA; main effect of FC *F*(1,12) = 11.45, *P* = 0.0054]. Optical densities were normalized against β-actin and calculated as a % of their respective vehicle control. CIH, chronic intermittent hypoxia; NTS, nucleus tractus solitarius.

### CIH Activates NTS Astrocytes

GFAP plays a critical role in astrocyte-neuron interactions as well as communication between cells in the CNS. Expression and cellular distribution of GFAP increase dramatically when astrocytes are activated in response to injury or cellular stress (e.g., hypoxia). It has been hypothesized that astrocytes regulate NTS neurotransmission mainly through postsynaptic modulation by gliotransmitters, but also through presynaptic modulation of neurotransmitter release (40).

Double immunofluorescent labeling of NTS GlyR and GFAP was carried in tissue harvested from normoxic- and CIH-exposed rats. CIH was associated with changes in astrocyte morphology (Fig. 5*A*). To quantify the impact of CIH on GFAP expression in NTS astrocytes, the inhibitor of glial metabolism fluorocitrate (FC, 100 µM) was used. GFAP protein expression is shown in Fig. 5*B*. GFAP expression was normalized to β-actin and FC treatment was compared with the vehicle-treated control group and calculated as the percentage. GFAP in NTS astrocytes was decreased by FC in samples from normoxic and CIH rats (Fig. 5*C*). There was a significant effect of FC on GFAP abundance that was independent of CIH exposure [two-way ANOVA; main effect of drug treatment *F*(1,12) = 11.45, *P* = 0.0054].

### Glial Inhibition Attenuates CIH-Augmented Glycinergic Signaling

**Figure 6.**
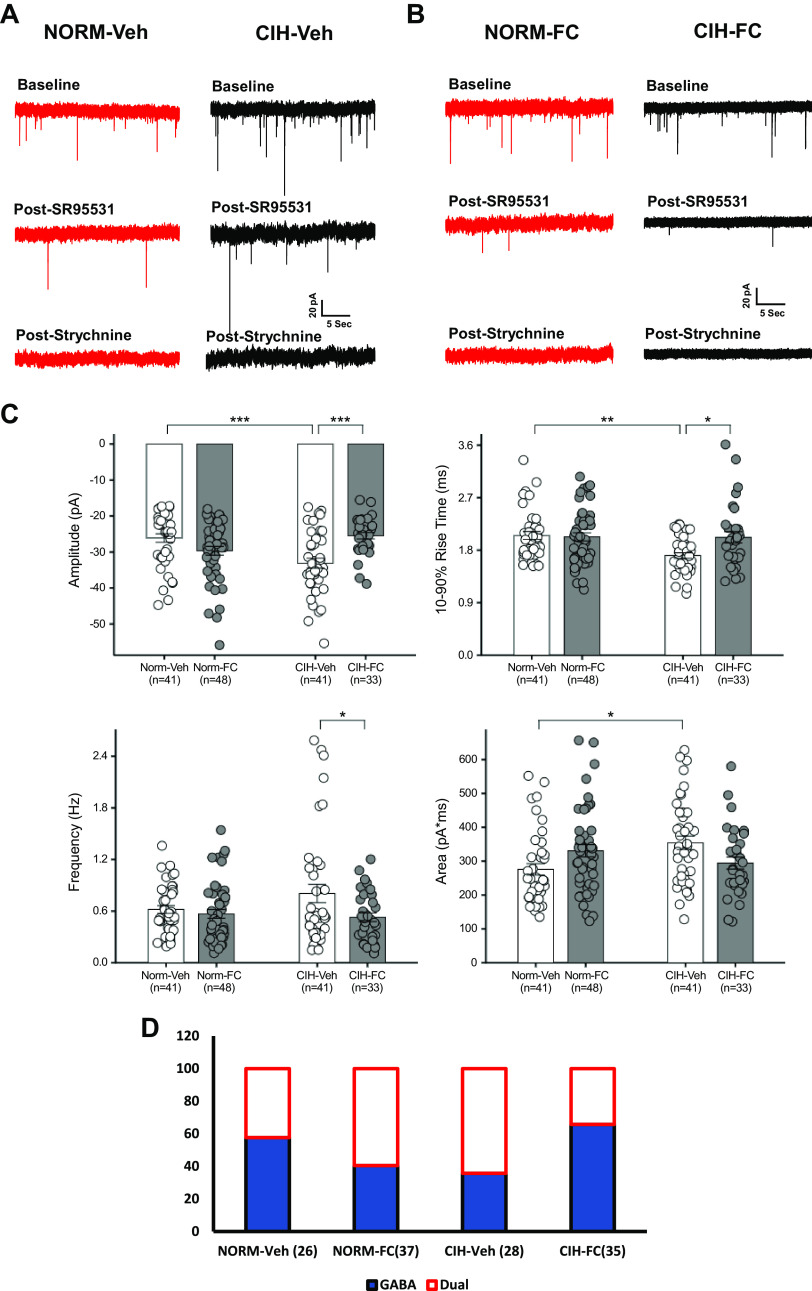
Inhibition of astrocyte activation reverses CIH upregulation of glycinergic mIPSCs. *A*: representative mIPSCs in slices pretreated with fluorocitrate (FC) or vehicle (Veh). No changes in mIPSC onset or decay kinetics were seen with Veh in NTS neurons from normoxia and CIH-exposed rats. *B*: representative mIPSCs in FC (100 µM)-treated slices showing amplitude and frequency were significantly reduced. *C*: mIPSC amplitude, area, frequency, and 10–90% rise time were compared between normoxic and CIH groups. Note that FC abolished all changes induced by CIH. *D*: in the CIH group, the proportion of NTS neurons expressing strychnine-sensitive mIPSCs was significantly decreased from 64.3% to 34.3% after FC treatment. By contrast FC had the opposite effect in the normoxic group, increasing GlyR mIPSCs from 42.3% to 59.5%. *P < 0.05. **P < 0.005. ***P < 0.001. CIH, chronic intermittent hypoxia; mIPSC, miniature inhibitory postsynaptic current; NTS, nucleus tractus solitarius. Each *n* represents the numbers of cells.

To examine the contribution of astrocytes in the increase of mIPSCs associated with CIH, brainstem slices containing the NTS were incubated with FC (100 µM) 2 h before and during the electrophysiology experiments. Representative recordings of putative second-order chemoreceptor NTS neurons from normoxic controls and CIH-conditioned rats indicate that unlike vehicle (Fig. 6*A*), FC appeared to prevent the increase in strychnine-sensitive mIPSCs associated with CIH (Fig. 6*B*).

FC also significantly affected other properties of mIPSCs in slices from CIH-conditioned rats. Pretreatment blocked effects of CIH on average mIPSC amplitude [*F*(3,159) = 7.9; *P* < 0.0001, CIH + Veh vs. CIH + FC, *P* = 0.0003; Fig. 6*C*] and rise time [*F*(3,159) = 3.8, *P* < 0.0114, CIH + Veh vs. CIH + FC, *P* < 0.03, Fig. 6*C*). These results suggest that FC reversed or attenuated the effects of CIH on the amplitude and onset kinetics of mIPSCs. In contrast, FC reduced mIPSC frequency in NTS neurons from CIH-treated rats [*F*(3,158) = 3.1, *P* < 0.0276, CIH + Veh vs. CIH + FC, Newman–Keuls test, *P* < 0.0368, Fig. 5*C*]. CIH increased mIPSC area significantly more in vehicle-treated slices from CIH rats than from normoxic controls [*F*(3,159) = 3.7, *P* < 0.0129, Norm + Veh vs. CIH +Veh, *P* = 0.0148; Fig. 6*C*].

In preparations exposed to the FC vehicle, CIH was associated with an increase in the numbers of strychnine-sensitive mIPSCs (Norm + Veh 11/26 vs. CIH + Veh 18/28 strychnine-sensitive mIPSCs; Fig. 6*D*) as previously observed. When preparations were exposed to FC, the number of strychnine-sensitive mIPSCs observed NTS neurons from CIH rats was comparable to that of normoxic controls (Norm + FC 22/37 vs. CIH + FC 12/35 neurons). This change produced by FC in the proportion of cells demonstrating strychnine-sensitive mIPSCs was statistically significant [χ^2^ (3) = 7.7, *P* = 0.0530].

## DISCUSSION

In the current study, we observed that 7 days of CIH was associated with increased glycine receptor-mediated mIPSCs in putative second-order chemoreceptor neurons in the caudal NTS. Greater GlyR contribution was reflected in mIPSCs displaying increased amplitude and faster onset kinetics compared with mIPSCs from cells that exhibited GABA mIPSCs. These effects were associated with increased GlyR immunoreactive puncta in NTS and they were blocked by pretreating brain slices with the protein trafficking inhibitor BFA. The BFA pretreatments prevented the increase in the numbers of NTS neurons demonstrating both GABA-AR and GlyR-mediated mIPSC following CIH, indicating that protein trafficking contributed to this effect. Collective results from confocal microscopy and BFA experiments suggest that CIH can enhance glycinergic neurotransmission in a subset of second-order NTS neurons receiving arterial chemoreceptor inputs. We cannot conclude that identified mIPSC changes are limited to this population of NTS neurons because unlabeled neurons were not studied. However, CIH did not influence mIPSCs in neurons from the DMNX suggesting that this is not a global effect. Similarly, CIH did not affect mIPSCs recorded from brain slice from female rats. As mentioned earlier, gonadally intact females do not show CIH hypertension associated with this 7-day protocol. Together these results suggest that the increase in GlyR-mediated mIPSCs following CIH is specific to the NTS of male rats and appears to be correlated with CIH hypertension.

Receptor trafficking involves scaffold proteins such as gephyrin, microtubule-associated motor proteins kinesin superfamily protein 5 (41), and dynein light chains 1 and 2 (42) in intracellular vesicles. This multiprotein complex is transported anterogradely and retrogradely between the neuronal cell body and distal neurites (43). BFA is a potent, reversible inhibitor of intracellular vesicle formation that reduces protein trafficking between the endoplasmic reticulum and the Golgi apparatus (44, 45). In previous studies, CIH has been shown to increase protein trafficking in several parts of the nervous system. For example, CIH has been shown to increase T-type calcium channel trafficking in the carotid body resulting in enhanced calcium influx associated with hypoxia (46). This effect of CIH was blocked by both BFA and a reactive oxygen species scavenger (46). In the pre-Bötzinger complex, CIH is associated with increased trafficking of 5-HT(2A) receptors, although mechanisms were not tested (47). In the NTS, CIH has been shown to increase AMPA receptor-mediated responses in acutely dissociated putative second-order chemoreceptor neurons (17). In contrast, CIH can also increase NTS excitation by decreasing excitatory amino acid transporter expression (18). The effects of CIH on NMDA receptor signaling in the NTS have been reported to either increase (16) or decrease (17). Collectively, available evidence indicates that CIH has complex effects on NTS neurotransmission that could be cell-type or CIH-protocol specific. Our results extend these observations by demonstrating that our model of CIH increases trafficking of GlyR leading to more diverse and rapid inhibitory responses. This change in NTS inhibitory neurotransmission could represent a homeostatic mechanism that opposes reported CIH increases in excitation (48). It could be speculated that such homeostatic compensations are why the hypertension in animal models of CIH is relatively modest (9, 34).

Previous studies have demonstrated an important role for astrocytes in NTS synaptic communication (27, 29, 49). Astrocytes help regulate the extracellular concentration of several neurotransmitters and have been shown to release several neuroactive amino acids such as taurine, serine, and glycine (49–52). Following CIH, we observed a qualitative increase in NTS astrocyte arborization based on GFAP staining and an associated increase in GlyR staining. However, Western blot analysis did not show a CIH-associated increase in GFAP abundance. This suggests that the CIH-related change in astrocyte morphology in caudal NTS was not reflected in a quantitative increase in astrocyte activation. Previous studies have shown that sustained hypoxia produces astrocyte activation in the NTS (30, 31) and that this effect is related to microglia activation (30). It could be that the CIH protocol used in our study was not sufficient to produce significant astrocyte activation as measured by GFAP abundance. It is also possible that the punch samples used for Western blot analysis contained parts of the NTS that were unaffected by CIH, preventing our ability to detect a significant increase in a subset of affected neurons. On this basis, additional experiments examining astrocyte morphology using Scholl analysis seem justified.

Based on these observations, we used FC to test the role of astrocytes in the CIH-related increase in GlyR-mediated mIPSC (53). FC inhibits the tricarboxylic acid cycle and preferentially affects astrocytes (53–55). This is thought to be due to the preferential tendency of astrocytes to take up and utilize acetate for cellular metabolism (56, 57). Metabolic inhibition of astrocytes by FC prevented or reversed the CIH-induced increase in the GlyR contribution to mIPSCs in putative second-order chemoreceptor NTS neurons. These findings are consistent with the role of astrocytes in gliotransmission in the NTS (49). In these studies, FC did not have significant effects in cells recorded from normoxic control rats even though our data show that FC decreased GFAP abundance in the NTS in samples from CIH-exposed rats and normoxic controls.

Glycine and serine are released both from neurons and astrocytes (58–60). In addition to its inhibitory effects, glycine is an important modulator of NMDA receptors (61). d-serine also functions as an agonist for the glycine binding sight on the NMDA receptor (61, 62). Release of d-serine and its binding to NMDA receptors contributes to activation of the respiratory network and could participate in central chemoreception (58). Astrocytes express glycine transporters and regulate the extracellular concentration of both glycine and serine (61). Our data suggest that some astrocytes in the NTS may express GlyRs and that CIH changes the morphology of some NTS astrocytes. Additional studies will be needed to determine how CIH influences these aspects of astrocyte function. Although our results indicate that GlyR expression is influenced by CIH, we cannot determine if CIH increased gliotransmission of glycine. If CIH does increase glycine or d-serine release from astrocytes, changes in NMDA receptor function might also be expected. Changes in excitatory responses of NTS neurons to NMDA and AMPA following CIH have been reported (16, 17). Whereas acute inhibition of astrocytes fails to alter the impact of CIH on respiration and sympathetic activity in juvenile rats, other studies suggest that NTS astrocytes are important contributors to basal neuronal function due to their role in the uptake of neural and glial transmitters (18, 28).

### Perspectives

Chronic intermittent hypoxia or CIH is a commonly used animal model pioneered by Fletcher and colleagues (2) to mimic the hypoxemias that occur in humans with sleep apnea. Protocols similar to the one used in the current study recapitulate key sequelae of sleep apnea such as hypertension and enhanced responses to hypoxia and heterotypic stressors (3, 4, 13, 63). Other CIH protocols produce beneficial effects (64) and therapeutic CIH is currently being used in clinical trials to improve motor function in patients with a variety of neuromuscular disorders (65). Both therapeutic intermittent hypoxia and CIH, as used to mimic sleep apnea in the current study, appear to produce neuroplasticity at multiple levels of the neural axis. Whether these changes are adaptive or maladaptive depends on if they contribute to or combat related pathology.

## GRANTS

This study was supported by grants P01 HL088052 and R01 HL155977 to G.E.F., G.M.T., and J.T.C.

## DISCLOSURES

No conflicts of interest, financial or otherwise, are declared by the authors.

## AUTHOR CONTRIBUTIONS

S.J., G.E.F., and J.T.C. conceived and designed research; S.J., N.R., K.K., and J.T.L. performed experiments; S.J., N.R., K.K., J.T.L., and J.T.C. analyzed data; S.J., N.R., K.K., G.E.F., J.T.L., G.M.T., and J.T.C. interpreted results of experiments; S.J., K.K., and G.M.T. prepared figures; S.J., G.M.T., and J.T.C. drafted manuscript; S.J., N.R., K.K., G.E.F., J.T.L., G.M.T., and J.T.C. edited and revised manuscript; S.J., N.R., K.K., G.E.F., J.T.L., G.M.T., and J.T.C. approved final version of manuscript.
